# Aquaporin-1 Facilitates Transmesothelial Water Permeability: In Vitro and Ex Vivo Evidence and Possible Implications in Peritoneal Dialysis

**DOI:** 10.3390/ijms222212535

**Published:** 2021-11-21

**Authors:** Francesca Piccapane, Andrea Gerbino, Monica Carmosino, Serena Milano, Arduino Arduini, Lucantonio Debellis, Maria Svelto, Rosa Caroppo, Giuseppe Procino

**Affiliations:** 1 Department of Biosciences, Biotechnologies and Biopharmaceutics, University of Bari, 70125 Bari, Italy; francesca.piccapane@uniba.it (F.P.); andrea.gerbino@uniba.it (A.G.); serena.milano@uniba.it (S.M.); lucantonio.debellis@uniba.it (L.D.); maria.svelto@uniba.it (M.S.); rosa.caroppo@uniba.it (R.C.); 2 Department of Sciences, University of Basilicata, 85100 Potenza, Italy; monica.carmosino@unibas.it; 3 Department of Research and Development, CoreQuest Sagl, 6900 Lugano, Switzerland; a.arduini@corequest.ch

**Keywords:** aquaporin 1, mesothelial cells, peritoneal dialysis, water transport, three-pore model

## Abstract

We previously showed that mesothelial cells in human peritoneum express the water channel aquaporin 1 (AQP1) at the plasma membrane, suggesting that, although in a non-physiological context, it may facilitate osmotic water exchange during peritoneal dialysis (PD). According to the three-pore model that predicts the transport of water during PD, the endothelium of peritoneal capillaries is the major limiting barrier to water transport across peritoneum, assuming the functional role of the mesothelium, as a semipermeable barrier, to be negligible. We hypothesized that an intact mesothelial layer is poorly permeable to water unless AQP1 is expressed at the plasma membrane. To demonstrate that, we characterized an immortalized cell line of human mesothelium (HMC) and measured the osmotically-driven transmesothelial water flux in the absence or in the presence of AQP1. The presence of tight junctions between HMC was investigated by immunofluorescence. Bioelectrical parameters of HMC monolayers were studied by Ussing Chambers and transepithelial water transport was investigated by an electrophysiological approach based on measurements of TEA^+^ dilution in the apical bathing solution, through TEA^+^-sensitive microelectrodes. HMCs express Zo-1 and occludin at the tight junctions and a transepithelial vectorial Na^+^ transport. Real-time transmesothelial water flux, in response to an increase of osmolarity in the apical solution, indicated that, in the presence of AQP1, the rate of TEA^+^ dilution was up to four-fold higher than in its absence. Of note, we confirmed our data in isolated mouse mesentery patches, where we measured an AQP1-dependent transmesothelial osmotic water transport. These results suggest that the mesothelium may represent an additional selective barrier regulating water transport in PD through functional expression of the water channel AQP1.

## 1. Introduction

The peritoneal membrane is considered as the first-line barrier that provides a protective, non-adhesive surface on the abdominal cavity and organs. The peritoneal membrane is structured by a layer of squamous-like and cuboidal ciliated mesothelial cells with microvilli and cell-cell junctional complexes, an interstitium containing bundles of collagen and mucopolysaccharides, and a dense network of capillaries, blood vessels, and lymphatics [1]. Mesothelial cells are mesodermal in origin and possess both epithelial and mesenchymal features, which allows for them to play numerous and important functions.

In addition to providing a protective and friction-free interface for the free movement of opposing organs and tissues, the mesothelium is involved in initiation and resolution of inflammation, tissue repair, release of factors to promote both the deposition and clearance of fibrin, and antigen presentation [2].

While this physiological role of the peritoneum was described long ago, more recently attention has focused on the possibility of exploiting the peritoneum as a selective membrane for performing PD. However, the role of mesothelial cells in mediating exchanges between blood capillaries and PD solutions is still unclear.

In the present work we wanted to address the question whether the mesothelial cells layer of the peritoneum may represent, in addition to endothelial cells, a second physical barrier to water diffusion in response to an osmotic gradient. This question is particularly relevant when considered in the context of PD.

PD is a kidney replacement therapy for patients with end-stage renal disease. In principle, the technique is based on the introduction into the patient’s peritoneal cavity of a hypertonic solution capable of attracting, through the peritoneal capillaries, excess water and toxins which the patient cannot eliminate due to impaired renal function. Considering the structure of the peritoneum, the pathway available for the water and solutes bidirectional diffusion from plasma to the PD solution includes the capillary endothelium, the sub-mesothelial interstitium, and the mesothelial layer [1,3]. According to the three-pore model [4,5,6,7], the major transport barrier in the peritoneum is the capillary endothelium. In particular, the water-selective “ultrasmall pores”, expressed on the endothelial cells, and later on identified as the water channels AQP1 [8,9,10], have been predicted to mediate half of the water ultrafiltration (UF) during crystalloid osmosis [5,6,11]. It has to be underlined that the three-pore model and most of the scientific literature on the PD field do not consider mesothelial cells of the peritoneum as a functional barrier regulating the rate of water diffusion in PD [11,12,13,14,15,16].

We previously reported that the water channel AQP1 is abundantly expressed in human peritoneal mesothelial cells, released through exosomes in the dialysis fluid during PD, and its abundance in the perfusate well correlates with dialysis efficiency [17]. The observation that mesothelial AQP1 strictly correlates with the amount of water UF and Na^+^ sieving during PD, suggests that mesothelial AQP1 significantly contributes to PD efficiency.

Here we focused on the functional role of AQP1 in facilitating water diffusion across the mesothelial cells, by recreating in vitro a mesothelial cells layer, sitting on extracellular matrix (ECM) layer, separating two different compartments at the apical and basolateral side. We used as experimental model an immortalized cell line, HMC, derived from human peritoneal mesothelium [18] that was characterized for the presence of tight junctions, the polarized and functional expression of plasma membrane Na^+^ transporters and transcellular water transport, either in the presence or in the absence of AQP1.

We mimicked the osmotic gradient generated upon infusion of the PD solutions in the peritoneal cavity, by adding the crystalloid osmolyte mannitol at different osmolarity in the apical compartment. We also tested glucose-based and glucose-free dialytic solutions currently used in clinical practice. We developed a method for measuring the transmesothelial water transport, by exploiting the dilution of an impermeable solute, TEA^+^, present in the apical compartment at a known concentration [19]. We also compared the results obtained in vitro, on mouse mesentery patches, freshly isolated from mouse mesenteric window.

Our results indicate that the presence of AQP1 in mesothelial cells is associated with an increase in the rate of osmotic water transport, thus suggesting that AQP1 may play the same role in mesothelium in vivo. This would imply that the regulation of the expression of mesothelial AQP1 in patients undergoing PD might have a great potential application in PD practice.

## 2. Results

### 2.1. Characterization of HMC

In this study we used an immortalized human mesothelial cell line HMC [18] characterized by a polygonal morphology when cultured on porous cell culture inserts coated with a layer of ECM in order to mimic the composition of the submesothelial interstitium. We evaluated the polarization and the barrier integrity of HMC monolayers by immunofluorescence analysis and by electrophysiological measurements respectively.

#### 2.1.1. Immunofluorescence Confocal Analysis

**Figure 1:**
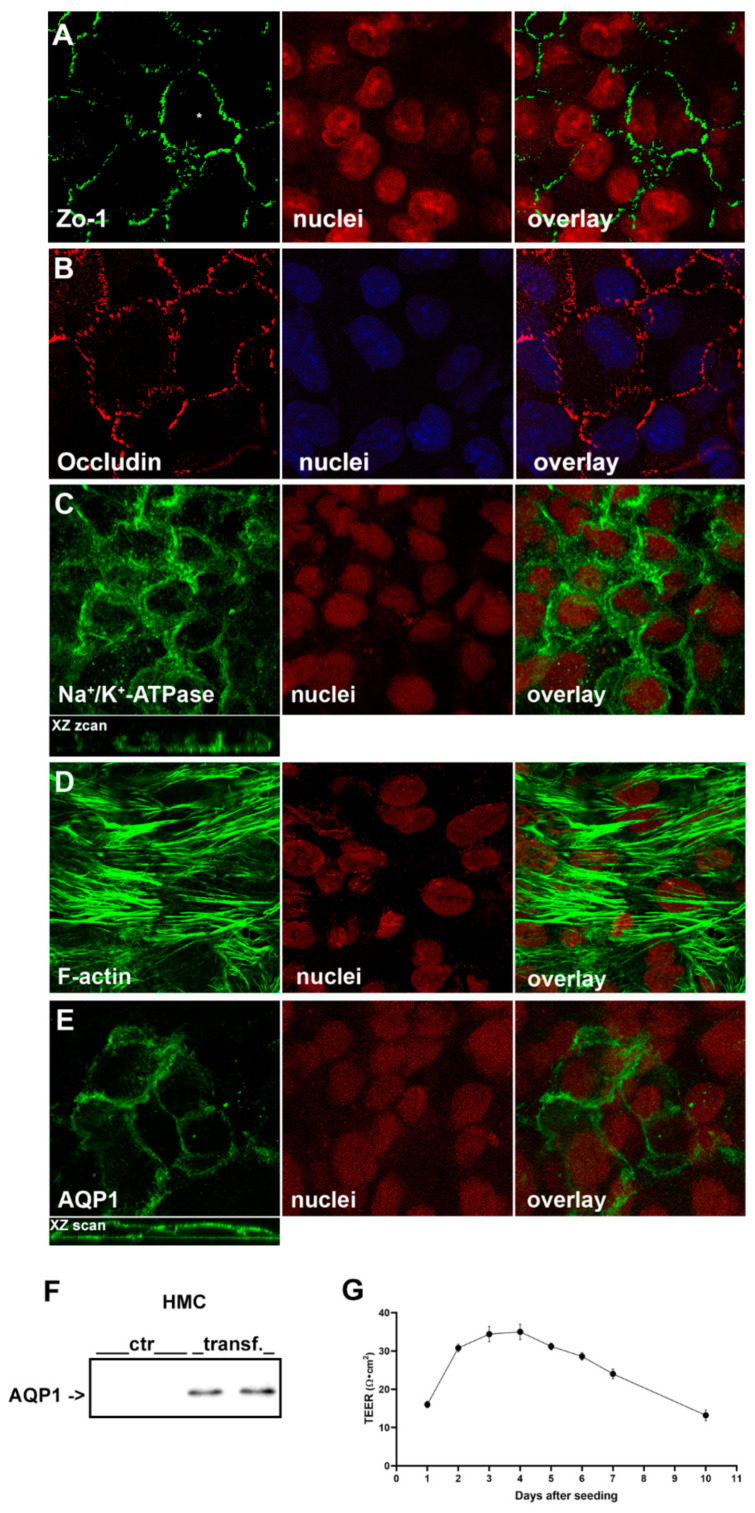
Characterization of HMC monolayers by immunofluorescence analysis and by TEER. HMC confluent monolayers, grown of porous inserts, were fixed in cold methanol and subjected to immunofluorescence. (**A**,**B**) Immunolocalization of the tight junction proteins anti Zo-1 and occludin. Both proteins decorated the cell-cell borders; (**C**) In confocal sections Na^+^/K^+^-ATPase was visible at the basolateral plasma membrane (see XZ scan); (**D**) Actin cytoskeleton was visualized by incubating HMC monolayers with fluorescent phalloidin; (**E**) Ectopic expression of transfected human AQP1 in HMC was visualized by monoclonal anti AQP1 localized both at apical and basolateral plasma membrane (see XZ scan); (**F**) Western blotting analysis of AQP1 expression in HMC, before (ctr) and after transfection with human AQP1 cDNA; (**G**) Time-course of TEER changes in HMC cultures. TEER values (Ω·cm^2^) at each time-point are expressed as mean values ± SEM of *n* = 5 monolayers.

Cells were subjected to immunofluorescence analysis to assess their ability to form in vitro a continuous layer of mesothelial cells. Figure 1A shows the expression and localization of the tight junction scaffolding protein zonula occludens-1 (Zo-1) at the cell contour, as obtained by confocal laser-scanning microscopy, thus suggesting that the cell layer is polarized. Antibodies against occludin, another junctional component known to directly interact with Zo-1, also indicated the formation of tight junctions (Figure 1B).

HMC exhibited also clear expression of the Na^+^/K^+^-ATPase at the basolateral plasma membrane (Figure 1C and XZ projection of the staining). Actin-based cytoskeleton was visualized by fluorescent phalloidin, showing well organized F-actin stress fibers within the cytoplasm of HMC (Figure 1D), indicating the presence of cell-substrate and cell-cell contacts [20].

HMC did not express endogenous AQP1 (Figure 1F), therefore we transfected them with human AQP1 cDNA. Anti AQP1 antibodies detected the protein at the plasma membrane (Figure 1E). Confocal scanning in the XZ plan confirmed the expression of AQP1 at both the apical and basolateral membrane of transfected HMC, in agreement with what we previously demonstrated in human mesothelial cells in vivo [17].

#### 2.1.2. Evaluation of HMC Monolayers Polarization

The integrity of HMC monolayers and the presence of junctional complexes were assessed by measuring the transepithelial electrical resistance (TEER). As shown in Figure 1G, the TEER across the HMC monolayers started to increase after 1 day of culture and the maximum value (35 ± 2.02 Ω·cm^2^, *n* = 5) was achieved between day 3 and 4, after which the TEER progressively decreased. Accordingly, we performed the next series of experiments on HMC monolayers at the day 4. At full confluence, M1-CCD cells reached a maximum of 657 ± 70.88 Ω·cm^2^ (*n* = 9).

To evaluate the barrier function of the HMC layers, the electrophysiological properties of the monolayers were studied by Ussing chambers. After an equilibration time of 15 min, baseline transepithelial potential (V_t_) was 0.6 ± 0.2 mV; short-circuit current (I_sc_) was 23.6 ± 8.1 μA/cm^2^; resistance (R_t_) was 26.9 ± 1.9 Ω/cm^2^ and the conductance (G_t_) was 38.7 ± 3.1 mS/cm^2^ (*n* = 9). These electrical values, although small, were consistently observed and indicate that HMC cells grown on filters form confluent and functional monolayers capable of maintaining a spontaneous transepithelial potential.

#### 2.1.3. Electrophysiological Characterization of HMC Monolayers

**Figure 2:**
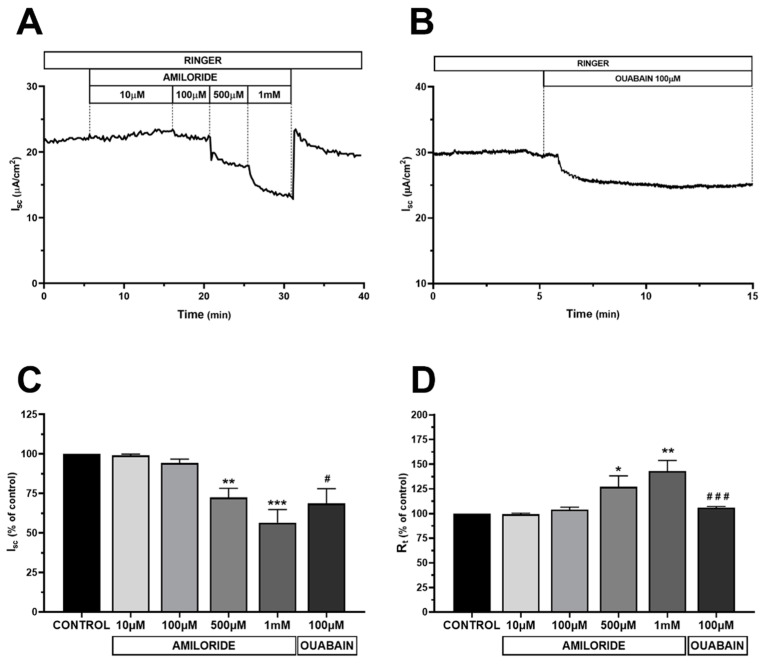
Functional polarization of HMC monolayers. (**A**) Representative trace of short circuit current (I_sc_) showing that apical amiloride dose-dependently decreased I_sc_ in HMC; (**B**) Representative trace recording of I_sc_ demonstrating that basolateral addition of ouabain (100 µM) in the bath solution induced a sustained decrease of I_sc_; (**C**,**D**) Changes in I_sc_ and R_t_, induced by Na^+^ channels inhibition (*n* = 4) and by Na^+^/K^+^-ATPase inhibition (*n* = 3). Results are expressed as percentage of the I_sc_ and R_t_ basal value; Values are reported as mean values ± SEM. * *p* <0.05, ** *p* < 0.01, *** *p* < 0.001 vs. basal condition calculated by one-way analysis of variance (ANOVA) with Dunnett’s multiple comparison test. # *p* < 0.05, ### *p* < 0.001 vs. basal condition calculated by Student t-test.

Since the immunofluorescence data suggested that HMC monolayers are polarized, the functional polarity of HMC was further investigated by short circuit current (I_sc_) measurements to test the vectorial Na^+^ transport across HMC monolayers. In epithelial cells, the unidirectional transport of Na^+^ is mediated by apical entry via amiloride-sensitive epithelial Na^+^ channels and basolateral extrusion by the ouabain-sensitive Na^+^ pumps. As reported in Figure 2A,C,D, addition of increasing concentrations of amiloride at the apical side of HMC monolayer, rapidly decreased I_sc_ and increased R_t_ when applied at 500 µM and 1 mM (−28% and −43% for I_sc_ and +27% and +43% for R_t_ compared to baseline, respectively). The amiloride removal led to a complete return of I_sc_ to the basal value. Of note, addition of amiloride at the basolateral side did not show any significant effect (data not shown).

This suggests that mesothelial HMC express amiloride-sensitivity -Na^+^ channels at the apical membrane as previously reported by others [21,22].

Inhibition of the Na^+^/K^+^-ATPase induced by addition of ouabain (100 µM) at the basolateral solution evoked a slow and significant reduction of I_sc_ (−31% of the initial value) and a slight increase of R_t_ (+6% of the initial value) (Figure 2B–D). Addition of ouabain at the apical side did not show any significant effect (not shown).

Along with the results of the immunofluorescence analysis, these data demonstrated that HMC can form a functional polarized monolayer and would represent a suitable experimental model for studying the transmesothelial water transport.

### 2.2. Real Time Measurements of Transmesothelial Water Flux across the HMC Monolayers and Mouse Mesentery: Role of AQP1

After checking the effect of TEA^+^ and mannitol on the electrophysiological parameters (see Figures S1 and S2, Supplemental Results [23,24,25]), we exploited TEA^+^-sensitive microelectrodes to investigate the water transport across HMC monolayers, in the presence or in the absence of transfected AQP1.

**Figure 3:**
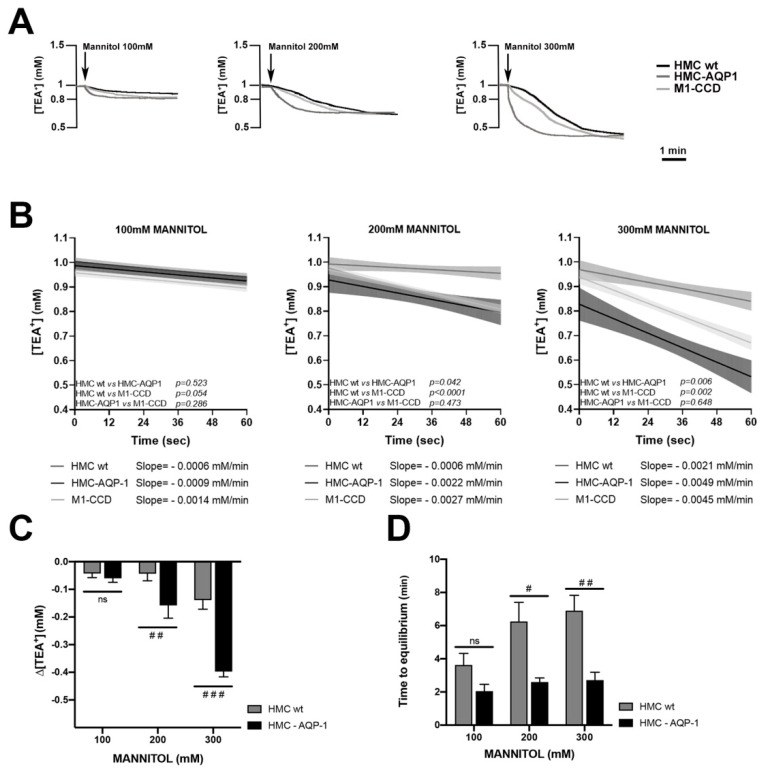
Osmotically induced transepithelial water transport in wt and AQP1-transfected HMC monolayers. (**A**) Representative traces of TEA^+^ concentration drop in response to increasing doses of mannitol as recorded by TEA^+^ sensitive microelectrodes; (**B**) Slopes (solid lines) and 95% confidence interval (shaded areas, CI) for the regression of [TEA^+^] calculated during the first 60 s of the response. Statistical analysis of the differences between slopes is reported within each panel. The value of the slopes is reported on the bottom of each panel; (**C**) Changes of [TEA^+^] (Δ[TEA^+^]) measured 1 min after application of mannitol in the apical bath; (**D**) Histogram representing the time required to reach maximal TEA^+^ dilution following mannitol addition. Data are reported as mean values ± SEM. # *p* < 0.05; ## *p* < 0.01; ### *p* < 0.001; vs. HMC wt calculated by two-way analysis of variance (ANOVA) with Sidak’s multiple comparison test. (Mann 100 mM: M1-CCD: *n* = 10; HMC wild type: *n* = 8; AQP1-transfected HMC: *n* = 4). (Mann 200 mM: M1-CCD: *n* = 7; HMC wild type: *n* = 6; AQP1-transfected HMC: *n* = 5). (Mann 300 mM: M1-CCD: *n* = 7; HMC wild type: *n* = 10; AQP1-transfected HMC: *n* = 7).

We also used renal M1-CCD cells [26,27] as a model of a tight epithelium not expressing endogenous water channels [28] in which the transepithelial water flux should be low. Figure 3A reports representative traces indicating the time- and mannitol concentration-dependent reduction of [TEA^+^] in HMC monolayers formed by wild type (black line) or AQP1-transfected monolayers (dark gray line) and renal M1-CCD monolayers (light gray line). Each curve shows that the initial concentration of apical TEA^+^ (1 mM) decreases in response to the water flux from the basolateral compartment until it reaches a steady state corresponding to the maximal dilution rate. The curves indicate, at each mannitol concentration added, a faster TEA^+^ dilution rate in HMC-AQP1 compared with wild type HMC.

To highlight the contribution of AQP1 to speed up the transepithelial water transport, we calculated the initial rate of decrease (slope) of the TEA^+^ dilution during the early phase of the response.

The initial interval of 1 min was arbitrary chosen and six time points, every 12 s, were analyzed on each curve. Figure 3B reports the slopes of non-linear regression between [TEA^+^] and time in wt HMC, AQP1-transfected HMC and renal M1-CCD cells, after adding different concentrations of mannitol.

Interestingly, the slopes of the curves observed in AQP1-transfected HMC at 200 and 300 mM mannitol, indicated a faster kinetic of TEA^+^ dilution compared to wt HMC, suggesting that AQP1 facilitates the transmesothelial water transport in the early phase (the slope with 200 mM mannitol in wt HMC was −0.0006 ± 0.0008 mM/min; in HMC-AQP1 = −0.0022 ± 0.0014 mM/min; the slope at 300 mM mannitol wt HMC was −0.0021 ± 0.0010 mM/min; in HMC-AQP1 = −0.0049 ± 0.0018 mM/min). Of note, in the absence of AQP1, HMC showed a slope of TEA^+^ dilution that was even slower than that calculated in M1-CCD renal cells, which also do not express AQP1.

Furthermore, we analyzed the onset of reduction of [TEA^+^], 1 min after the addition of the mannitol in the apical chamber. As reported in Figure 3C, the presence of AQP1 in HMC was associated with a significant higher dilution of [TEA^+^] (three- to four-folds) in the first minute, compared with wt HMC, at least when creating an osmotic gradient with 200 and 300 mM mannitol.

Then, we calculated the time response for the maximal dilution of TEA^+^, either in the presence or in the absence of AQP1 (Figure 3D). Interestingly, in the presence of AQP1, the maximal dilution of [TEA^+^] in the apical compartment was reached in half the time compared to untransfected monolayers and the difference was statistically significant, at least after addition of 200 and 300 mM mannitol.

When we measured the maximal dilution of TEA^+^, achieved at each mannitol concentration, we did not observe differences between the three cell lines, thus indicating that all cell lines allow the same maximal dilution of TEA^+^, although with different kinetics (data not shown). Altogether, these data confirmed that in HMC, AQP1 represents an additional pathway for transmesothelial water transport in response to an osmotic gradient.

**Figure 4:**
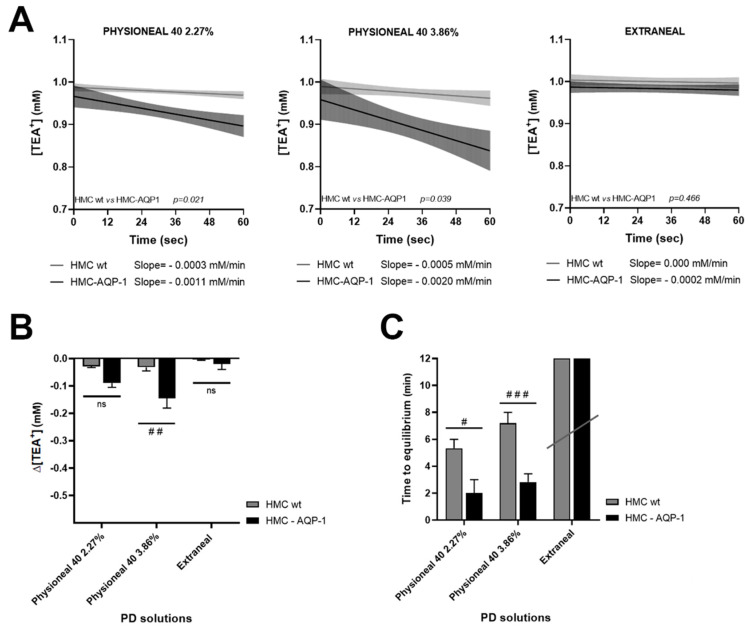
Osmotically induced transepithelial water transport in wt and AQP1-transfected HMC monolayers in response to Physioneal 40 2.27%, Physioneal 40 3.86% and Extraneal. (**A**) Slopes (solid lines) and 95% confidence interval (shaded areas, CI) for the regression of [TEA^+^] calculated during the first 60 s of the response. Statistical analysis of the differences between slopes is reported within each panel. The value of the slopes is reported on the bottom of each panel; (**B**) Changes of [TEA^+^] (Δ[TEA^+^]) measured 1 min after application of PD solutions in the apical bath; (**C**) Histogram representing the time required to reach maximal TEA^+^ dilution following PD solutions addition. Data are reported as mean values ± SEM. # *p* < 0.05; ## *p* < 0.01; ### *p* < 0.001; vs. HMC wt calculated by two-way analysis of variance (ANOVA) with Sidak’s multiple comparison test. (Physioneal 2.27%: HMC wild type: *n* = 3; AQP1-transfected HMC: *n* = 3). (Physioneal 3.86%: HMC wild type: *n* = 5; AQP1-transfected HMC: *n* = 6). (Extraneal: HMC wild type: *n* = 3; AQP1-transfected HMC: *n* = 3).

We also tested the effect of common peritoneal dialysis solutions, used in clinical settings: Physioneal 40 (Baxter), containing 2.27% and 3.86% of the crystalloid osmolyte glucose, and Extraneal (Baxter), containing 7.5% of the colloid osmolyte icodextrin. Figure 4A reports the initial rate of decrease (slope) of the TEA^+^ dilution during the early phase of the response to Physioneal 40 (2.27%), Physioneal 40 (3.86%), and Extraneal in wt HMC cells (grey line), and AQP1-transfected HMC (dark line).

Interestingly, in analogy with the results obtained using mannitol, the slopes of the curves observed in AQP1-transfected HMC exposed at the apical side to 2.27% and 3.86% glucose-based Physioneal solutions, indicated a statistically significant faster kinetic of TEA^+^ dilution compared to wt HMC, confirming the role of AQP1 in facilitating transmesothelial water transport induced by crystalloid osmolytes. Conversely, using the icodextrin-based Extraneal solution, supposed to elicit a slow, AQP1-independent, water movement through colloid osmosis, we did not measure statistically significant differences between the slopes obtained in HMC in the presence or in the absence of AQP1 (the slope with Physioneal 2.27% in wt HMC was −0.0003 ± 0.0002 mM/min; in HMC-AQP1 = −0.0011 ± 0.0007 mM/min; the slope with Physioneal 3.86% in wt HMC was −0.0005 ± 0.0005 mM/min; in HMC-AQP1 = −0.0020 ± 0.0013 mM/min; the slope with Extraneal in wt HMC was 0.000 ± 0.000 mM/min; in HMC-AQP1 = −0.0002 ± 0.0007 mM/min).

Figure 4B indicates that the presence of AQP1 was associated with a significant higher dilution of [TEA^+^] (three- to four-folds) in the first minute, in HMC, at least when exposing cells to Physioneal 40 with the higher osmotic strength (3.86% glucose). As the osmotic response to icodextrin was very slow, in the first minute after exposure to Extraneal we detected a very small dilution of [TEA^+^] and no difference was attributable to the presence of AQP1 in HMC.

In agreement with experiments performed with mannitol, using glucose as crystalloid osmotic agent, in the presence of AQP1, the maximal dilution of [TEA^+^] in the apical compartment was reached in half the time compared to untransfected monolayers and the difference was statistically significant (Figure 4C). Using icodextrin as osmotic agent, the slower kinetic of water transport did not allow to reach an equilibrium, at least after 20 min of observation (Figure 4C, Extranel).

**Figure 5:**
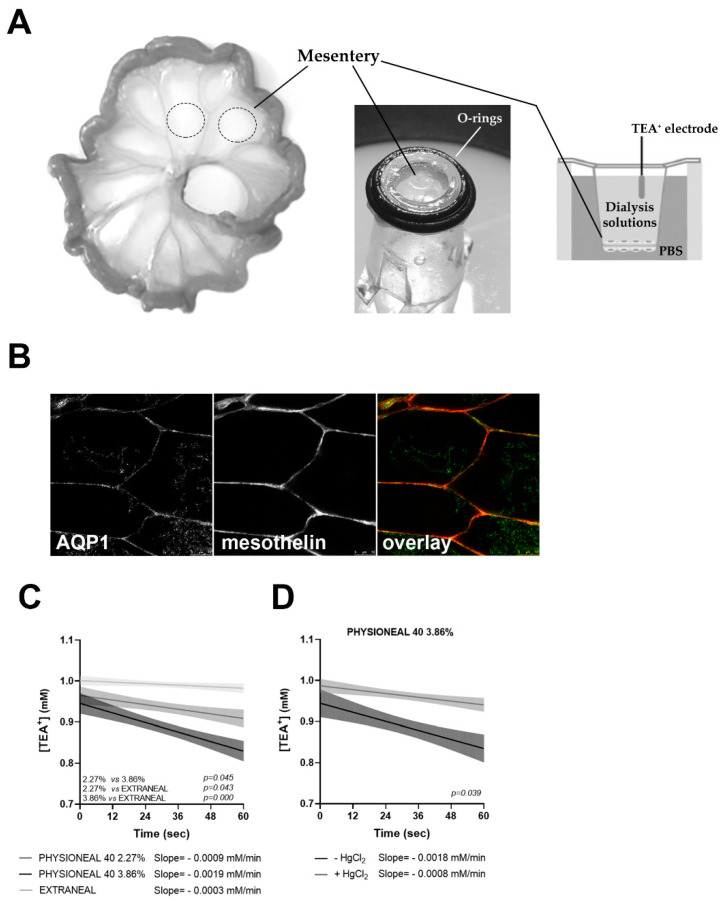
Transepithelial water transport in the mouse mesentery and role of AQP1. (**A**) Schematic representation of isolation of mouse mesentery patches for measurements of the osmotically induced transcellular water flux with TEA^+^-sensitive microelectrodes; (**B**) Characterization of mouse mesentery by immunofluorescence analysis: in confocal sections AQP1 and mesothelin co-localized at the plasma membrane. bar = 10 µm; (**C**) Osmotically induced transepithelial water transport in mouse mesentery in response to Physioneal 40 2.27%, Physioneal 40 3.86% and Extraneal. Slopes (solid lines) and 95% confidence interval (shaded areas, CI) for the regression of [TEA^+^] calculated during the first 60 s of the response. Statistical analysis of the differences between slopes is reported within each panel. The value of the slopes is reported on the bottom of each panel; (Physioneal 2.27%: *n* = 3; Physioneal 3.86%: *n* = 5; Extraneal: *n* = 3); (**D**) Osmotically induced transepithelial water transport in mouse mesentery in response to Physioneal 40 3.86%**,** in the presence or in the absence of HgCl_2_. The value of the slopes is reported on the bottom of each panel (Physioneal 3.86% without HgCl_2_: *n* = 3; Physioneal 3.86% with HgCl_2_: *n* = 3).

Next, we thought to validate these results on animal tissue ex vivo. The simplest system, consisting of two sheets of peritoneum fused together, is represented by the mesentery. As reported in Figure 5A, small intestine was isolated from adult mice soon after sacrifice and small mesentery patches (dotted circles) were sealed, with the aid of O-rings, on transwell supports on which the porous membrane was excised. The double sheet of mesothelium was subjected to measurements of TEA^+^ dilution, as previously described, by placing the dialysis solutions (Physioneal 40 and Extraneal) in the upper chamber. In these conditions, as there is no blood circulation inside the capillaries and small vessels, the contribution of the endothelium can be considered negligible and water can only move from the lower to the upper compartment, crossing the two layers of mesothelium. Confocal scanning confirmed the co-localization of AQP1 and mesothelin at the plasma membrane of mouse mesentery (Figure 5B).

Interestingly, as reported in Figure 5C, the slopes of the curves observed in mouse mesentery after incubation with Physioneal 2.27% and 3.86%, indicated a faster kinetic of TEA^+^ dilution compared to Extraneal, suggesting that AQP1 facilitates the transmesothelial water transport induced by crystalloid osmotic agents in the early phase (the slope with Physioneal 2.27% in mouse mesentery was −0.0009 ± 0.0006 mM/min; the slope with Physioneal 3.86% was −0.0019 ± 0.0007 mM/min; the slope with Extraneal was −0.0003 ± 0.0003 mM/min).

Of note, as shown in Figure 5D, in the presence of HgCl_2_ at 500 μM, an AQP1 inhibitor, the slope of TEA^+^ dilution was significantly slower than that calculated in the absence (the slope with Physioneal 3.86% without HgCl_2_ was −0.0018 ± 0.0009 mM/min and with HgCl_2_ was −0.0008 ± 0.0005 mM/min).

## 3. Discussion

Since the discovery of the AQP1 water channel in 1992 [29], scientists have sought to understand the function of the 13 aquaporins cloned to date, in the various tissues where they are expressed. In addition to maintaining cell volume, rapid water exchange across cells enables tissues and organs to secrete and/or absorb water as part of their physiological function. AQP1 is detected in the endothelium lining peritoneal capillaries, venules, and small veins [30] and we previously reported a clear expression of AQP1 in human peritoneal mesothelial cells [17]. The physiological function of AQP1 in mesothelial cells of the peritoneum could be to secrete water into the peritoneal cavity which, together with glycosaminoglycans and lubricants, facilitates intracoelomic movement of organs. In recent decades, with the increase in the clinical practice of PD, it has been understood that, although in a non-physiological context, the presence of AQP1 in the peritoneal capillaries is a key element ensuring the dialysis process [11].

Currently, the widely accepted model that describes water and solute transport during PD is represented by the ‘three-pore model’, a computer-based simulation elaborated by Rippe and Stelin [4,5,6,7].

The three-pore model assumes that the only limiting barrier regulating the major transport in the peritoneum is the capillary endothelium which contains, indeed, three types of pores: (a) the ‘small pores’, corresponding to clefts between endothelial cells, with an average radius of 40–50 Å, accounting for the 95% of the hydraulic conductivity (L_p_S); (b) the ‘large pores’ represented by venular interendothelial gaps, average radius 250 Å, accounting for 5% of the L_p_S; (c) the ‘ultra-small’, water-selective pores associated with the plasma membrane of endothelial cells, radius 3–5 Å, accounting for only the 1–2% of the transcellular L_p_S. The latter, was subsequently identified as the water channel aquaporin-1 (AQP1) [8,10,29], and since they reject solutes but only facilitate water transport, they mediate half of the water UF during a crystalloid osmosis, as it occurs during a dwell with hypertonic glucose [11]. Later on, Flessner and Rippe implemented the three-pore-model taking into account the barrier effect of the matrix of fibers in the interstitium [31]. Even in this novel computational model, a possible role of mesothelial cells as a third functional barrier to water movement during PD was not considered. Yet, the abundant expression of AQP1 on human mesothelial cells [17], and the fact that, the hydraulic conductivity (L_p_S) of the mesothelium is comparable to that of the endothelium of the peritoneal capillaries [32] would suggest that the transport of water through the mesothelium, exploits the transcellular rather than the paracellular pathway, and, therefore, it may be strictly dependent on the availability of AQP1. Indeed, we showed a positive correlation between the amount of AQP1 released in the PD effluent by mesothelial cells and UF, free water transport and Na^+^-sieving [17].

We exploited an immortalized mesothelial cell line of human peritoneal origin due to the limitation of primary cell lines which already start to transform at the third passage and did not survive beyond passage six in culture [33]. In our cell culture conditions, we found that mesothelial cells form a continuous, polarized cell layer. In fact, HMC showed strong Zo-1 tight junctional bands at the cell-cell contacts and express the tetraspan TJ protein occludin, an important determinant for the regulation of paracellular permeability [34], which is responsible for sealing intercellular TJs [35]. This evidence suggests that the presence of occludin might reduce the paracellular route for water movement. Of note, endogenous expression of TJ proteins Z0-1 and occludin in human peritoneal mesothelial cells has been previously reported [36,37,38] and their role in limiting the paracellular passage of water and solutes has been demonstrated in bovine retinal endothelial cells [39] and in cultured human peritoneal mesothelial cells [40]. In our experiments, the presence of an intact and continuous junctional barrier in HMC was also confirmed by increase of TEER over time.

Epithelial cell polarity is essential for the establishment and maintenance of vectorial transport of ions and fluids that provides the basis for appropriate reabsorptive and secretory function. The Na^+^/K^+^-ATPase is expressed in mesothelial cells of human peritoneum [41], sheep visceral and parietal pleura [42,43], in rabbit pleura [44] and according to our immunohistological study, it is also expressed in the plasma membrane of HMC (Figure 1C). Interestingly, basolateral but not apical, addition of ouabain (100 µM), significantly reduced the I_sc_ and increased R_t_, as assessed by voltage clamp technique. The existence of amiloride-sensitive apical Na^+^ conductance was previously shown by Ussing experiments in mesothelial cells of human parietal peritoneum, sheep visceral peritoneum, human and sheep parietal pleura [22]. In our hands, exposure of the apical, but not basolateral, membrane of HMC to amiloride had significant effect on the measured bioelectrical parameters, I_sc_ and R_t_. Altogether, these findings in HMC indicated a vectorial transepithelial transport of Na^+^ and are consistent with the establishment of a polarized monolayer suitable for studying the role of AQP1 water channel in the transmesothelial water transport.

The lack of endogenous expression of AQP1 in HMC is not surprising as it is quite common that, in particular for AQPs, the lack of osmotic challenge in culture condition can downregulate AQPs expression. In fact, it has been reported by several groups that some AQPs, including AQP1, are regulated via osmotic response elements and hypertonicity [45,46,47,48]. The lack of endogenous expression of AQP1 in HMC gave us the opportunity to test the rate of transmesothelial water transport in the absence and in the presence of AQP1, given that, besides HgCl_2_ which is highly toxic to cells, specific drugs that inhibit AQPs function are still lacking.

The low values of TEER measured in HMC indicated that they form a “leaky” cell monolayer, in terms of ion transport, compared to classic tight epithelia, such as the renal collecting duct. However, HMC confluent monolayers do not behave as a leaky epithelium in terms of transepithelial water flow. In fact, the analysis of the slopes of the TEA^+^ dilution curves, in response to the transepithelial water flux (Figure 3B), indicated that, in the absence of AQP1, the dilution rate of TEA^+^ in HMCs was even lower than that of a monolayer of renal cells showing high TEER values. This could be due to the fact that the renal cells used, being murine, and therefore smaller than HMC, would express a larger area occupied by cell-cell junctions which represent a path for the paracellular movement of water. Strikingly, though, transient transfection of HMCs with AQP1, which mesothelial cells express in vivo [17], increased the dilution rate of TEA^+^ by two to three times, suggesting that the presence of AQP1 increases transepithelial water transport.

We confirmed our findings with both mannitol and glucose as osmotic agents. Interestingly, icodestrin-based PD solution, which generate a slow water flow that predominantly occurs across small pores and is independent of tonicity [49,50], elicited an AQP1-independent response in HMCs. This suggests that the mesothelium behave as the capillary endothelium in terms of selectivity to water diffusion during PD.

In addition, we performed water transport experiments on mouse mesentery, formed by a double layer of visceral peritoneum and endogenously expressing AQP1 on mesothelial cells [51]. Experiments confirmed the results obtained in cultured HMCs: we recorded a fast and AQP1-dependent (HgCl_2_-inhibible) osmotic water transport in the presence of the crystalloid osmolytes glucose and a slow water transport in the presence of the colloid osmolytes icodextrin. Both results clearly suggest that the crystalloids-elicited osmotic water transport across mesothelial cells in vivo might depend on the presence of AQP1. Indeed, the abundant expression of AQP1 in mesothelial cells in vivo might indicate that these cells need a water channel at the plasma membrane to speed up transcellular water transport, because the paracellular pathway is not per se sufficient to guarantee adequate flow.

It is possible that the minor importance attributed so far to the mesothelium as a functional barrier toward the passage of water may be due, in some cases, to artifacts of the conventional fixation procedure, that induce loss of mesothelial cells, cells shrinkage, and appearance of intercellular gaps [52], erroneously suggesting that the mesothelium could not be a functional barrier to the passage of water.

In conclusion, these data suggest that: (1) in vivo the mesothelium could represent a limiting barrier controlling the transcellular diffusion of water from the submesothelial interstitium to the peritoneal cavity during PD with crystalloid osmolytes and that AQP1 facilitates this process; (2) HMCs can be considered a good in vitro model to study transmesothelial transport phenomena. From a translational point of view our data open a scenario of possible interventions to make the dialysis process more efficient and extend the dialysis life of patients with end-stage renal disease. A number of drugs have been shown to upregulate AQP1 expression in patients [53,54,55,56]. In addition, the notion that AQP1 is upregulated by hypertonicity [48] but downregulated by glucose degradation products [57], contained in conventional glucose-based PD solutions, suggests that new solutions with osmotic agents other than glucose, as the one we recently proposed [18], in addition to being more biocompatible [58], could better preserve the integrity of mesothelium and its AQP1 content, thus resulting in more efficient water UF.

## 4. Materials and Methods

### 4.1. Chemicals

Amiloride hydrochloride hydrate (Amiloride, #A7410), Barium chloride (BaCl_2_, #342920), Tetraethylammonium chloride (TEA^+^, #T2265), Ouabain (#O3125), D-Mannitol (Mannitol, #M4125), Mercury chloride (HgCl_2_, #215465), propidium iodide (#P4170), were purchased from Sigma Aldrich (St. Louis, MO, USA); all stock solutions were prepared in water and stored at −20 °C until use.

Antibodies: goat anti-Occludin (N-19), (cat. #sc-8145, dilution 1:100), mouse anti-AQP1 (β-11), (cat. #sc-25287, dilution 1:100), mouse anti-Mesothelin (C-2), (cat. #sc-365324, dilution 1:100), were from Santa Cruz Biotechnology, Dallas, TX, USA; anti-Na^+^/K^+^-ATPase α-1 antibody was from Merk (Burlington, MA, USA); mouse monoclonal anti-ZO-1, (ZO1-1A12) was from Thermo Fisher Scientific (Waltham, MA, USA). 

### 4.2. Cell Culture

The Immortalized Human Mesothelial Cells-SV40 (HMC) were purchased from Applied Biological Materials (ABM, Richmond, BC, Canada) and cultured as previously described [18].

The M1 cortical collecting duct cell line (M1-CCD) [27] was obtained from ATCC (www.lgcstandards-atcc.org, accessed on 21 October 2021) and cultured as elsewhere described [59].

For immunofluorescence and water transport experiments, HMC and M1-CCD were grown on 0.4 μm pore size permeable Transwell™ supports (0.9 cm^2^) (#353180, Corning, Glendale, AZ, USA), while for Ussing chamber experiment we used Snapwell™ supports with 1.12 cm^2^ cell growth area (#3801). Both cell lines were seeded at a density of 2.2 × 10^5^ cells/cm^2^ on filters previously coated with ECM (cat. #G422, ABM, Richmond, BC, Canada).

HMC monolayers were transiently transfected with the human AQP1 plasmid pRP[Exp]-Neo-CMV>hAQP1 [NM_198098.2] (VectorBuilder, Neu-Isenburg, Germany), using lipofectamine 2000 reagent (Thermo Fisher Scientific, Waltham, MA, USA), according to the manufacturer’s protocol. For immunofluorescence or water transport experiments, monolayers were processed 48 h after transfection.

### 4.3. Transepithelial Electrical Resistance Measurements

The integrity of the cell monolayer was routinely tested by measuring TEER with the Epithelial Voltohmmeter EVOM 2 (World Precision Instruments, Sarasota, FL, USA) according to manufacturer’s protocol and as previously discussed [18].

### 4.4. Immunofluorescence and Confocal Microscopy

For immunofluorescence experiments, HMC monolayers or mesentery patches were fixed in cold methanol (−20 °C) for 5 min and washed twice with phosphate-buffered saline (PBS, pH 7.4). Cells were incubated in 1% bovine serum albumin in PBS for 30 min at room temperature to block unspecific binding sites for the following antibodies: mouse anti-Na^+^/K^+^-ATPase α-1 antibody, (cat. #05-369 clone C464.6, dilution 1:500), mouse monoclonal anti-ZO-1, (ZO1-1A12), (cat. #33-9100, dilution 1:1000), goat anti-Occludin (N-19), (cat. #sc-8145, dilution 1:100), mouse anti-AQP1 (β-11), (cat. #sc-25287, dilution 1:100), mouse anti-Mesothelin (C-2), (cat. #sc-365324, dilution 1:100), overnight at 4 °C in blocking buffer. After three washes in PBS, cells were incubated with 555 or 488 Alexafluor-conjugated secondary antibodies and Alexafluor 488 Phalloidin (cat. #A12379, dilution 1:500; Thermo Fisher Scientific) for 1 hr at room temperature.

After washes in PBS, nuclei were stained with propidium iodide (500 nM) according to the manufacturer’s protocol. Confocal pictures were taken with a Leica SP5 microscope.

### 4.5. Ussing Chamber Studies

Transepithelial measurements were performed on HMC grown as monolayers for 4–5 days. Inserts were mounted onto Easy Mount Ussing chambers (Physiologic Instruments Inc., Reno, NV, USA).

Apical and basolateral hemichambers were filled with 5 mL of Ringer’s solutions containing in mM: 117.15 NaCl, 25 NaHCO_3_, 1.15 NaH_2_PO_4_, 5.65 KCl, 1.2 MgSO_4_, 1.2 CaCl_2_, 5 glucose (pH 7.4, 300 mOsm). The solution was gassed with a 95% O_2_—5% CO_2_ mixture and constantly recirculated by gas bubble lift.

The short circuit current (I_sc_) and the transepithelial resistance (R_t_) were measured with a VCC-MC4 voltage clamp amplifier (Physiologic Instruments Inc., Reno, NV, USA) using two pairs of Ag/AgCl electrodes connected to the chamber solutions through 3% agar bridges containing 3 M KCl.

Offset of the electrodes pairs and fluid electrical resistance were evaluated prior to the onset of each experiment and nullified so that they are not included in the determination of electrical measurements.

Bipolar current pulse of 1 µA amplitude and 200 ms duration was applied every 60s to the monolayer and the R_t_ was automatically calculated from the change in open-circuit voltage according to Ohm’s law.

HMC monolayers were allowed to equilibrate for 15 min prior to the experiments being performed. After reaching a steady state I_sc_ values, specific blockers of different ion transporters were added. Data were collected using the Acquire and Analyze program, version 2.3 (Physiologic Instruments Inc., Reno, NV, USA).

### 4.6. Transmesothelial Water Flux Measurements

Real time measurements of water flux across the HMC monolayers or mouse mesentery were performed using an electrophysiological approach previously developed and based on the dilution of the cell-impermeant TEA^+^, using TEA^+^-sensitive microelectrodes [19].

**Figure 6:**
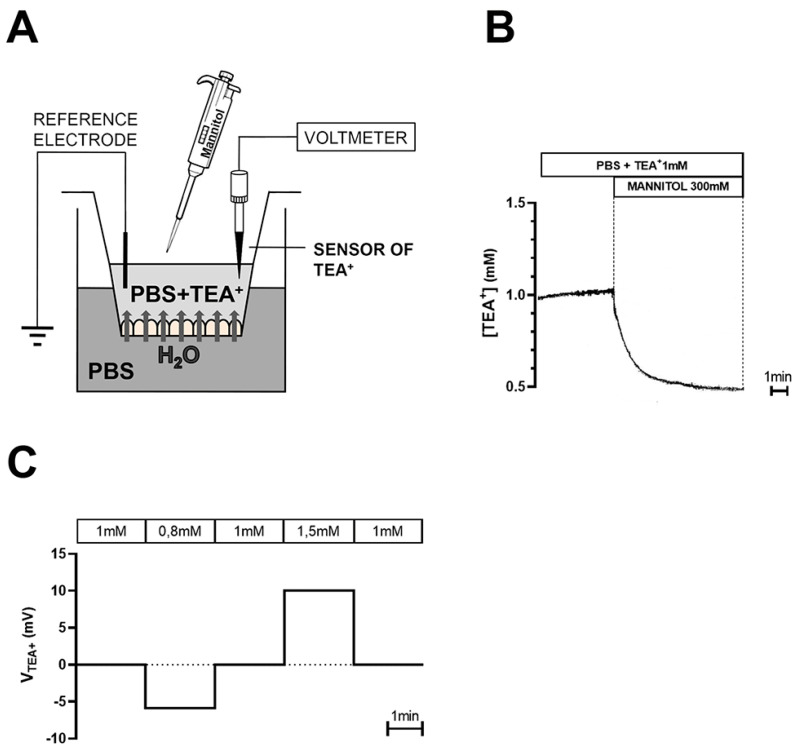
Protocol for transepithelial water transport measurements in HMC monolayers with TEA^+^-sensitive microelectrodes. (**A**) Schematic representation of set-up used to measure the osmotically induced transcellular water flux across HMC monolayers with TEA^+^-sensitive microelectrodes positioned in the apical compartment; (**B**) Addition of 300 mM mannitol in the apical chamber triggered a transepithelial water flux from the basolateral chamber that decreased [TEA^+^] recorded by the TEA^+^-sensitive microelectrode in a renal cell monolayer; (**C**) Calibration of TEA^+^-sensitive microelectrodes by exposure to PBS solutions containing different [TEA^+^].

Briefly, the basolateral compartment of Transwell inserts was filled with 1 mL of PBS, while the apical compartment, bathing the TEA^+^-sensitive electrode, was filled with 200 µL of PBS containing 1 mM TEA^+^ (Figure 6A).

The water flux was induced by adding the osmotic agent mannitol (100, 200, and 300 mM) in the apical compartment containing TEA^+^, thus allowing a water flux from the basolateral compartment and, consequently, a drop in TEA^+^ concentration in the apical compartment (Figure 6B). In a different set of experiments, peritoneal dialysis solutions were added in the apical compartment after being supplemented with 1 mM TEA^+^. We used Physioneal 40 (2.27% and 3.86% glucose, from Baxter) and Extraneal (7.5% Icodextrin, from Baxter). Microelectrodes were calibrated before and after each experiment in PBS solutions containing different TEA^+^ concentrations (0.8, 1.0, and 1.5 mM, see Figure 6C) and were discarded when the measured slope was not within 10% of the calculated slope. The average slope of the electrodes was 58.37 ± 1.0 mV for a 10-fold change in TEA^+^ concentration (*n* = 23).

TEA^+^-sensitive microelectrodes were constructed as reported in Supplemental Methods. Changes in TEA^+^ concentration were monitored in the apical solution bathing HMC monolayer or mouse mesentery, by lowering perpendicularly the ion-sensitive microelectrode, mounted on a Leitz micromanipulator, in the close proximity of the monolayer under oblique (45°) observation through a stereomicroscope at 50× magnification.

All measurements were performed with a model FD 223 dual channel electrometer (World Precision Instruments, Sarasota, FL, USA) and recorded on a strip chart recorder.

### 4.7. Data Analysis and Statistics

GraphPad Prism version 8.0 (GraphPad Software, San Diego, CA, USA) was used for the statistical analysis and graph representation of the electrophysiological data. Data are given as mean value ± standard error of *n* individual recordings.

Statistical analysis of data was carried out by using Student’s *t* test for paired data or the one-way or two-way analysis of variance (ANOVA) with Dunnett’s or Sidak’s multiple comparison test when appropriate. A *p* value < 0.05 was considered statistically significant.

For calculation of the slopes, the data recorded in the first minute, every 12 s, was fit to a line using non-linear regression analysis. This analysis uses the linear least square fitting method to find the best fitting line for a given set of data points by minimizing the sum of squares of the offsets.

Data were shown as slope ± 95% confidence interval (CI) of the slope for the non-linear regression between TEA^+^ concentration and time of *n* individual recordings.

The extra sum-of-squares F test was used to evaluate the differences in the best-fit parameter of slope among data sets (100, 200, 300 mM mannitol) in the three cell lines according to the software’s recommended approach; differences were considered significant for *p* < 0.05.

## Supplementary Materials

The following are available online at https://www.mdpi.com/article/10.3390/ijms222212535/s1.

## Abbreviations

PD	Peritoneal Dialysis
AQP1	Aquaporin1
UF	Ultrafiltration
TEER	TransEpithelial Electrical Resistance
ECM	Extracellular matrix
HMC	Human Mesothelial Cells

## References

[1] Devuyst O., Rippe B. (2014). Water transport across the peritoneal membrane. Kidney Int..

[2] Mutsaers S.E. (2002). Mesothelial cells: Their structure, function and role in serosal repair. Respirology.

[3] Mehrotra R., Devuyst O., Davies S.J., Johnson D.W. (2016). The current state of peritoneal dialysis. J. Am. Soc. Nephrol..

[4] Rippe B., Levin L. (2000). Computer simulations of ultrafiltration profiles for an icodextrin-based peritoneal fluid in CAPD. Kidney Int..

[5] Rippe B., Stelin G., Haraldsson B. (1991). Computer simulations of peritoneal fluid transport in CAPD. Kidney Int..

[6] Rippe B., Venturoli D., Simonsen O., de Arteaga J. (2004). Fluid and electrolyte transport across the peritoneal membrane during CAPD according to the three-pore model. Perit. Dial. Int..

[7] Stelin G., Rippe B. (1990). A phenomenological interpretation of the variation in dialysate volume with dwell time in CAPD. Kidney Int..

[8] Agre P., Preston G.M., Smith B.L., Jung J.S., Raina S., Moon C., Guggino W.B., Nielsen S. (1993). Aquaporin CHIP: The archetypal molecular water channel. Am. J. Physiol. Physiol..

[9] Jung J.S., Preston G.M., Smith B.L., Guggino W.B., Agre P. (1994). Molecular structure of the water channel through aquaporin CHIP. The hourglass model. J. Biol. Chem..

[10] Nielsen S., Smith B.L., Christensen E.I., Agre P. (1993). Distribution of the aquaporin CHIP in secretory and resorptive epithelia and capillary endothelia. Proc. Natl. Acad. Sci. USA.

[11] Ni J., Verbavatz J.-M., Rippe A., Boisdé I., Moulin P., Rippe B., Verkman A.S., Devuyst O. (2006). Aquaporin-1 plays an essential role in water permeability and ultrafiltration during peritoneal dialysis. Kidney Int..

[12] Zhang W., Freichel M., Van Der Hoeven F., Nawroth P.P., Katus H., Kälble F., Zitron E., Schwenger V. (2016). Novel endothelial cell-specific AQP1 knockout mice confirm the crucial role of endothelial AQP1 in ultrafiltration during peritoneal dialysis. PLoS ONE.

[13] Devuyst O., Goffin E. (2008). Water and solute transport in peritoneal dialysis: Models and clinical applications. Nephrol. Dial. Transplant..

[14] Rippe B., Krediet R.T. (1994). Peritoneal physiology-transport of solutes. The Textbook of Peritoneal Dialysis.

[15] Marples D. (2001). Aquaporins: Roles in renal function and peritoneal dialysis. Perit. Dial. Int..

[16] Schoenicke G., Diamant R., Donner A., Roehrborn A., Grabensee B., Plum J. (2004). Histochemical distribution and expression of aquaporin 1 in the peritoneum of patients undergoing peritoneal dialysis: Relation to peritoneal transport. Am. J. Kidney Dis..

[17] Corciulo S., Nicoletti M.C., Mastrofrancesco L., Milano S., Mastrodonato M., Carmosino M., Gerbino A., Corciulo R., Russo R., Svelto M. (2019). AQP1-containing exosomes in peritoneal dialysis effluent as biomarker of dialysis efficiency. Cells.

[18] Piccapane F., Bonomini M., Castellano G., Gerbino A., Carmosino M., Svelto M., Arduini A., Procino G. (2020). A novel formulation of glucose-sparing peritoneal dialysis solutions with L-carnitine improves biocompatibility on human mesothelial cells. Int. J. Mol. Sci..

[19] Gerbino A., Fistetto G., Colella M., Hofer A.M., Debellis L., Caroppo R., Curci S. (2007). Real time measurements of water flow in amphibian gastric glands: Modulation via the extracellular Ca2+-sensing receptor. J. Biol. Chem..

[20] Brückner B.R., Nöding H., Skamrahl M., Janshoff A. (2019). Mechanical and morphological response of confluent epithelial cell layers to reinforcement and dissolution of the F-actin cytoskeleton. Prog. Biophys. Mol. Biol..

[21] Nie H.G., Tucker T., Su X.F., Na T., Peng J.B., Smith P.R., Idell S., Ji H.L. (2009). Expression and regulation of epithelial Na+ channels by nucleotides in pleural mesothelial cells. Am. J. Respir. Cell Mol. Biol..

[22] Ji H.-L., Nie H.-G. (2008). Electrolyte and fluid transport in mesothelial cells. J. Epithel. Biol. Pharmacol..

[23] Neher E., Lux H.D. (1973). Rapid changes of potassium concentration at the outer surface of exposed single neurons during membrane current flow. J. Gen. Physiol..

[24] Nicholson C. (1993). Ion-selective microelectrodes and diffusion measurements as tools to explore the brain cell microenvironment. J. Neurosci. Methods.

[25] Stanfield P.R. (1983). Tetraethylammonium ions and the potassium permeability of excitable cells. Rev. Physiol. Biochem. Pharmacol..

[26] Fejes-Toth G., Naray-Fejes-Toth A. (1992). Differentiation of renal β-intercalated cells to α-intercalated and principal cells in culture. Proc. Natl. Acad. Sci. USA.

[27] Stoos B.A., Náray-Fejes-Tóth A., Carretero O.A., Ito S., Fejes-Tóth G. (1991). Characterization of a mouse cortical collecting duct cell line. Kidney Int..

[28] Mancinelli R., La Rovere R.M.L., Fulle S., Miscia S., Marchisio M., Pierdomenico L., Lanuti P., Procino G., Barbieri C., Svelto M. (2014). Extracellular GTP is a potent water-transport regulator via aquaporin 5 plasma-membrane insertion in M1-CCD epithelial cortical collecting duct cells. Cell. Physiol. Biochem..

[29] Preston G.M., Carroll T.P., Guggino W.B., Agre P. (1992). Appearance of water channels in Xenopus oocytes expressing red cell CHIP28 protein. Science.

[30] Devuyst O., Ni J. (2006). Aquaporin-1 in the peritoneal membrane: Implications for water transport across capillaries and peritoneal dialysis. Biochim. Biophys. Acta Biomembr..

[31] Rippe B., Venturoli D. (2007). Simulations of osmotic ultrafiltration failure in CAPD using a serial three-pore membrane/fiber matrix model. Am. J. Physiol.-Ren. Physiol..

[32] Breborowicz A., Knapowski J. (1986). Transmesothelial ultrafiltration in vitro. Perit. Dial. Bull..

[33] Stylianou E., Jenner L.A., Davies M., Coles G.A., Williams J.D. (1990). Isolation, culture and characterization of human peritoneal mesothelial cells. Kidney Int..

[34] Furuse M., Hirase T., Itoh M., Nagafuchi A., Yonemura S., Tsukita S., Tsukita S. (1993). Occludin: A novel integral membrane protein localizing at tight junctions. J. Cell Biol..

[35] Hirase T., Staddon J.M., Saitou M., Ando-Akatsuka Y., Itoh M., Furuse M., Fujimoto K., Tsukita S., Rubin L.L. (1997). Occludin as a possible determinant of tight junction permeability in endothelial cells. J. Cell Sci..

[36] Ito T., Yorioka N., Kyuden Y., Asakimori Y., Kiribayashi K., Ogawa T., Kohno N. (2003). Effect of glucose polymer on the intercellular junctions of cultured human peritoneal mesothelial cells. Nephron. Clin. Pract..

[37] Horiuchi T., Matsunaga K., Banno M., Nakano Y., Nishimura K., Hanzawa C., Miyamoto K.I., Nomura S., Ohta Y. (2009). HPMCs induce greater intercellular delocalization of tight junction-associated proteins due to a higher susceptibility to H_2_O_2_ compared with HUVECs. Perit. Dial. Int..

[38] Retana C., Sanchez E., Perez-Lopez A., Cruz A., Lagunas J., Cruz C., Vital S., Reyes J.L. (2015). Alterations of intercellular junctions in peritoneal mesothelial cells from patients undergoing dialysis: Effect of retinoic acid. Perit. Dial. Int..

[39] Antonetti D.A., Wolpert E.B., DeMaio L., Harhaj N.S., Scaduto R.C. (2002). Hydrocortisone decreases retinal endothelial cell water and solute flux coincident with increased content and decreased phosphorylation of occludin. J. Neurochem..

[40] Kaneda K.I., Miyamoto K., Nomura S., Horiuchi T. (2006). Intercellular localization of occludins and ZO-1 as a solute transport barrier of the mesothelial monolayer. J. Artif. Organs.

[41] Witowski J., Breborowicz A., Topley N., Martis L., Knapowski J., Oreopoulos D.G. (1997). Insulin stimulates the activity of Na+/N+-ATPase in human peritoneal mesothelial cells. Perit. Dial. Int..

[42] Hatzoglou C.H., Gourgoulianis K.I., Molyvdas P.A. (2001). Effects of SNP, ouabain, and amiloride on electrical potential profile of isolated sheep pleura. J. Appl. Physiol..

[43] Agostoni E., Zocchi L. (1990). Solute-coupled liquid absorption from the pleural space. Respir. Physiol..

[44] Zocchi L., Agostoni E., Cremaschi D. (1991). Electrolyte transport across the pleura of rabbits. Respir. Physiol..

[45] Herrlich A., Leitch V., King L.S. (2004). Role of proneuregulin 1 cleavage and human epidermal growth factor receptor activation in hypertonic aquaporin induction. Proc. Natl. Acad. Sci. USA.

[46] Jenq W., Cooper D.R., Bittle P., Ramirez G. (1999). Aquaporin-1 Expression in Proximal Tubule Epithelial Cells of Human Kidney Is Regulated by Hyperosmolarity and Contrast Agents. Biochem. Biophys. Res. Commun..

[47] Umenishi F., Schrier R.W. (2002). Identification and characterization of a novel hypertonicity-responsive element in the human aquaporin-1 gene. Biochem. Biophys. Res. Commun..

[48] Umenishi F., Schrier R.W. (2003). Hypertonicity-induced aquaporin-1 (AQP1) expression is mediated by the activation of MAPK pathways and hypertonicity-responsive element in the AQP1 gene. J. Biol. Chem..

[49] Leypoldt J.K., Mistry C.D. (1994). Ultrafiltration in peritoneal dialysis. The Textbook of Peritoneal Dialysis.

[50] Morelle J., Sow A., Fustin C.-A., Fillée C., Garcia-Lopez E., Lindholm B., Goffin E., Vandemaele F., Rippe B., Öberg C.M. (2018). Mechanisms of crystalloid versus colloid osmosis across the peritoneal membrane. J. Am. Soc. Nephrol..

[51] Wang J., Wang Y., Lou Y., Cui W., Zhang Y., Dong W., Sun J., Miao L. (2021). Effect of aquaporin 1 on mouse peritoneal mesothelial cells after a long-term peritoneal dialysis. Ther. Apher. Dial..

[52] Liu S.M., Li J., Wang Y., Ye R.G., Lindholm B., Wang T. (2001). Methods to improve the preservation of peritoneal tissues. Adv. Perit. Dial..

[53] Kobayashi H., Yokoo H., Yanagita T., Satoh S., Kis B., Deli M., Niwa M., Wada A. (2006). Induction of aquaporin 1 by dexamethasone in lipid rafts in immortalized brain microvascular endothelial cells. Brain Res..

[54] Guan Y., Chen J., Zhan Y., Lu H. (2018). Effects of dexamethasone on C6 cell proliferation, migration and invasion through the upregulation of AQP1. Oncol. Lett..

[55] Xu J., Huang B., Wang Y., Tong C., Xie P., Fan R., Gao Z. (2016). Emodin ameliorates acute lung injury induced by severe acute pancreatitis through the up-regulated expressions of AQP1 and AQP5 in lung. Clin. Exp. Pharmacol. Physiol..

[56] Dong C., Wang G., Li B., Xiao K., Ma Z., Huang H., Wang X., Bai C. (2012). Anti-asthmatic agents alleviate pulmonary edema by upregulating AQP1 and AQP5 expression in the lungs of mice with OVA-induced asthma. Respir. Physiol. Neurobiol..

[57] Lin X., Amore A., Loiacono E., Balegno S., Manniello D., Peruzzi L., Camilla R., Minieri V., Daprà V., Qian J. (2009). Effect of glucose degradation products, glucose-containing dialysate and icodextrin on AQP1 and eNOS expression in cultured endothelial cells. J. Nephrol..

[58] Bonomini M., Zammit V., Divino-Filho J.C., Davies S.J., Di Liberato L., Arduini A., Lambie M. (2021). The osmo-metabolic approach: A novel and tantalizing glucose-sparing strategy in peritoneal dialysis. J. Nephrol..

[59] Iolascon A., Aglio V., Tamma G., D’Apolito M., Addabbo F., Procino G., Simonetti M.C., Montini G., Gesualdo L., Debler E.W. (2006). Characterization of two novel missense mutations in the AQP2 gene causing nephrogenic diabetes insipidus. Nephron Physiol..

