# Supplementary material for: Aquaporin-1 Facilitates Transmesothelial Water Permeability: In Vitro and Ex Vivo Evidence and Possible Implications in Peritoneal Dialysis
Source: Int J Mol Sci. 2021 Nov 21;22(22):12535. doi: 10.3390/ijms222212535 (PMC8622642; doi:10.3390/ijms222212535)
Supplement: Supplementary file 1 [file ijms-22-12535-s001.zip › ijms-1453266-supplementary.pdf]

Supplementary material

Aquaporin-1 Facilitates Transmesothelial Water Permeability: In Vitro and Ex Vivo Evidence and Possible Implications in Peritoneal Dialysis

Francesca Piccapane [1], Andrea Gerbino [1], Monica Carmosino [2], Serena Milano [1], Arduino Arduini [3], Lucantonio Debellis [1], Maria Svelto [1], Rosa Caroppo [1#] and Giuseppe Procino [1#,*].

[1] Department of Biosciences, Biotechnologies and Biopharmaceutics, University of Bari, Italy; francesca.piccapane@uniba.it; andrea.gerbino@uniba.it; serena.milano@uniba.it; lucantonio.debellis@uniba.it; maria.svelto@uniba.it; rosa.caroppo@uniba.it; giuseppe.procino@uniba.it

[2] Department of Sciences, University of Basilicata, Potenza, Italy; monica.carmosino@unibas.it

[3] Department of Research and Development, CoreQuest Sagl, Technopole, Manno, Switzerland; a.arduini@corequest.ch

# Both authors have equally contributed to the manuscript.

* Correspondence: giuseppe.procino@uniba.it.

1. Supplemental methods

1.1. Construction of TEA^+ electrodes

TEA^+-sensitive microelectrodes were constructed from a piece of filament-containing aluminum silicate glass tubing of 1.5-mm outer diameter and 1.0-mm inner diameter (Hilgenberg, Malsfeld, Germany). Briefly, microelectrodes were pulled in a PE2 vertical puller (www.narishige-group.com), silanized for 90 s in dimethyldichlorosilane vapor (www.sigmaaldrich.com) and baked in the oven. The shank of the channel was back-filled with a small amount of the ligand mixture IE-190 (www.wpiinc.com), and its shaft was later filled with PBS and 1 mM TEA^+. The reference electrode was an Ag/AgCl wire connected to ground.

The ligand used to fill our microelectrodes is a K^+-selective liquid ion-exchanger that is about three orders of magnitude more sensitive to TEA^+ than to K^+ [1], therefore in the presence of high concentration of TEA^+ (1 mM) it becomes selective only to TEA^+. Although ion-selective microelectrodes respond to ionic activities and not to concentrations, calibrating solutions and results are reported as concentrations with the assumption that the activity coefficient does not change significantly during measurements [2].

2. Supplemental results

2.1. Possible effect of TEA^+ on apical K^+ conductance

TEA^+ is a known K^+ channel blocker with different degrees of efficacy [3], depending on the cellular model.

Thus, we were concerned that the presence of 1 mM TEA^+ in the apical solution of HMC monolayers during the measurements of water transport, could inhibit K^+ movement and consequently modify the physiological state of the HMC. To rule out this possibility, before performing our studies with TEA^+-sensitive microelectrodes, we carried out control experiments to test the effect of 1 mM TEA^+ on short circuit current.

As shown in supplemental Figure S1A, apical addition of 1 mM TEA^+ did not induce significant changes of the I_{sc} and the R_t. In the same experiments, we also used $BaCl_2$, a well-known inhibitor of different types of K^+ channels. The application of 1 mM $BaCl_2$ in the apical bath caused a rapid and reversible drop of I_{sc} (-26 % of the initial value, Figure S1B) and a significant increase of R_t (+58% of the initial value, Figure S1C) as a consequence of the apical K^+ conductance inhibition. Taken together, these observations indi-

cated that, although HMC express functional $BaCl_2$-sensitive K^+ channels at apical membrane, these are not affected by TEA^+, at least at the concentration intended for the water-flux experiments.

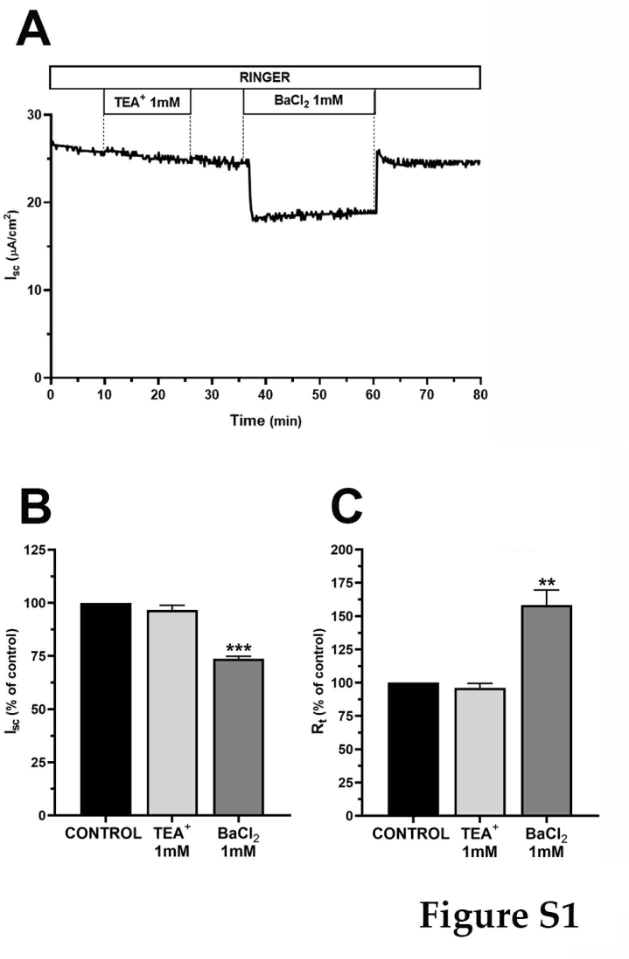

Figure S1. Apical K^+ channels are not inhibited by 1 mM TEA^+. (**A**) Representative trace recording of I_{sc} showing that apical addition of TEA^+ in the bath solution had no effect on the baseline I_{SC}. The subsequent apical application of $BaCl_2$ (1 mM) led to a strong decrease in I_{sc}. (**B**, **C**) Changes in I_{sc} and R_t changes induced by TEA^+ (n=6) and $BaCl_2$ (n=3). Results are expressed as percentage of the Isc and Rt basal value. Data are reported as mean values ± SEM ** p <0.01, *** p <0.001 vs basal condition calculated by calculated by one-way analysis of variance (ANOVA) with Dunnett's multiple comparison test.

2.2. Effect of mannitol on short circuit current and transepithelial resistance

We next checked for the possible effect of mannitol, the osmotic agent aimed to elicit water flux in our experiments, on the electrophysiological parameters of HMC monolayers. We therefore measured electrophysiological parameters in the same conditions intended for water flux experiments, namely adding 300 mM mannitol in the presence of 1 mM TEA^+.

As reported in Figure S2A, the presence of mannitol in the apical perfusion, did not significantly change the baseline I_{sc} and R_t (Figure S2B), suggesting that the ionic transports and the integrity of mesothelial monolayers are unaffected by the addition of mannitol.

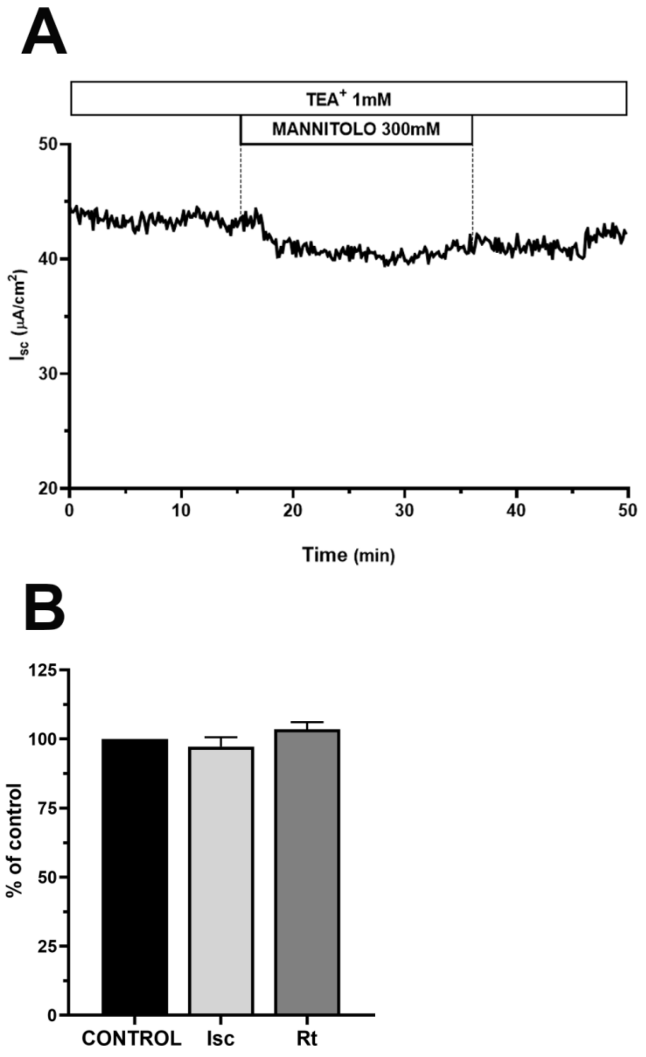

Figure S2

Figure S2. Effect of apical addition of mannitol (300 mM) on electrical parameters of HMC monolayers. (**A**) Representative time course of I_{sc} showing that apically applied mannitol, in the presence of 1 mM TEA^+, did not affect the baseline I_{sc}.; (**B**) Changes in I_{sc} and R_t induced by mannitol in the presence of TEA^+ (n=4). Results are expressed as percentage of the I_{sc} and R_t basal value. Data are reported as mean values ± SEM. Differences are not statistically significant by Student t-test.

References

1. Neher, E.; Lux, H.D. Rapid changes of potassium concentration at the outer surface of exposed single neurons during membrane current flow. *J. Gen. Physiol.* **1973**, *61*, 385–399, doi:10.1085/jgp.61.3.385.
2. Nicholson, C. Ion-selective microelectrodes and diffusion measurements as tools to explore the brain cell microenvironment. *J. Neurosci. Methods* **1993**, *48*, 199–213.
3. Stanfield, P.R. Tetraethylammonium ions and the potassium permeability of excitable cells. *Rev. Physiol. Biochem. Pharmacol.* **1983**, *97*, 1–67.
